# Digital Health Technology and the New Graduate Nurse: A Scoping Review Protocol

**DOI:** 10.3390/nursrep16030090

**Published:** 2026-03-05

**Authors:** Meagan Ryan, Victoria Cole, Judy Duchscher, Richard Booth, Michelle Lalonde

**Affiliations:** 1 School of Nursing, Faculty of Health Sciences, University of Ottawa, Ottawa, ON K1N 6N5, Canada; michelle.lalonde@uottawa.ca; 2 Rankin School of Nursing, St. Francis Xavier University, Antigonish, NS B2G 2W5, Canada; 3 Health Librarian, Faculty of Health Sciences, University of Ottawa, Ottawa, ON K1N 6N5, Canada; 4 School of Nursing, Thompson Rivers University, Kamloops, BC V2C 0C8, Canada; jduchscher@tru.ca; 5 Arthur Labatt Family School of Nursing, Western University, London, ON N6A 4V2, Canada; rbooth6@uwo.ca

**Keywords:** new graduate nurses, digital health, digital health technology, scoping review

## Abstract

Digital health technologies are being used in healthcare more than ever, which has implications for the daily work of nurses. As the newest members of the nursing profession, new graduate nurses (NGNs) experience great change during the transition to practice experience. The experience of NGNs transitioning to practice while digital health technologies are being increasingly integrated is not well elucidated in the nursing literature. This proposed scoping review will address this gap and aims to explore and describe the literature involving NGNs and digital health technologies. This review will use the Joanna Briggs Institute (JBI) guidelines to search CINAHL, MEDLINE, Embase, and ERIC databases for keywords and subject headings related to the concepts of “digital health technology” and “new graduate nurses”, published between 2020 and 2026. Included articles will involve new graduate nurses with 0–12 months of experience, use digital health technology in the clinical context of nursing, and be peer-reviewed primary research. Articles will be screened and extracted using Covidence and described in line with JBI guidance and presented narratively. The findings of this scoping review will be key in positioning the transition to practice experience for NGNs in an age of digital revolution. Results will be instrumental in enhancing nursing curriculum, ensuring transition policies and procedures are supportive of developing digital health competence and assuring the delivery of better care to patients when using digital health technologies. The contribution of this review will be unique and novel in exploring NGNs and digital health, providing context for the modern experience of transition to practice.

## 1. Introduction

Digital health technologies have been evolving, aligning with the way nurses care for their patients each day. From the integration of advanced technologies such as artificial intelligence to electronic medical records, there is a requirement for nurses to have knowledge and the ability to integrate technologies into the care of patients. Simultaneously, nurses are bearing unprecedented burdens, including a workforce shortage, thus signaling the importance of the recruitment and retention of new nurses. New graduate nurses (NGNs) experience complex shifts during the transition to practice experience. As a key cohort of registered nurses (RNs), NGNs hold a unique position in the nursing profession as the newest members of the profession, having recently graduated and offer fresh perspectives on nursing practice. As digital health technologies become more advanced and present in the daily work of nurses, it is vital to explore what is known about digital health technologies and NGNs.

## 2. Background

Digital health is defined as “the field of knowledge and practice associated with the development and use of digital technologies to improve health” [1] (p. 11). Furthermore, to reflect the position of the International Council of Nurses and Canadian Nurses’ Association on the advancement of technology, the terms “digital health” and “digital health technologies” will encompass specific technologies that relate closely to the current and future work of RNs outlined by these organizations. This includes technologies nurses currently use or are projected to be using in their daily practices, specifically: artificial intelligence, wearables, robotics, virtual care, and voice recognition software [2,3]. These technologies were specifically selected as they are anticipated to evolve and be used in nursing approaches to care of patients [2]. Technology use in nursing is not a novel phenomenon, but the emergence of COVID-19 accelerated the rate at which digital health technologies were being introduced and implemented in the health system and nursing roles [4]. Simultaneously, the need to acquire the knowledge and skill to integrate these technologies to provide patient care while protecting the privacy and safety of patients and assuring regulatory compliance represents a challenge for nurses [4]. This illustrates the need for nurses to achieve a degree of digital health competency to ensure that integration of knowledge into practice is occurring when technology is present [5].

Nursing students are challenged to meet entry-level competencies for digital health practice [6,7], but the experiences and abilities of NGNs are yet to be explored as it relates to these competencies. Understanding the experience of the NGN is imperative, as significant personal and professional changes occur in the process of transitioning from nursing student to RN [8,9]. While there are strategies to enhance the quality of digital health nursing education in baccalaureate programs, gaps persist in nursing education that leave nurses largely unprepared to enter practice [10].

The transition to practice experience is a non-linear and complex process [9]. It is important to distinguish the unique experience of the NGN during the transition to practice process in the digital context of the current health landscape. While there is a plethora of research published on the transition to nursing practice [11,12,13,14], there is a lack of understanding of how NGNs transition within the context of increasing digital health technology use. Given the empirical gap in the nursing literature at the intersection of NGNs and increasing technology in clinical settings, a scoping review is proposed to address this issue.

## 3. Methods

This scoping review uses the Joanna Briggs Institute (JBI) approach. The objective of this review is to explore and describe the literature about the use of digital health technologies on NGNs. The review question is, what is known in the literature about NGNs and digital health technologies? The population of interest is NGNs, the concept of digital health, and the context is the first year of practice.

The scoping review protocol has been registered in the Open Science Framework (OSF) and is currently under embargo. The OSF registration code is: osf.io/73r9h. Any deviations from this protocol will be reported in the final manuscript [13]. This review will follow the Preferred Reporting Items for Systematic Reviews and Meta-Analyses for Scoping Reviews checklist (PRIMSA-ScR).

### 3.1. Eligibility Criteria

Articles that will be included are primary research articles that use quantitative, qualitative or mixed methods and are peer-reviewed. Gray literature will be excluded from this scoping review as it does not align with the objective of exploring the peer-reviewed literature. Tool-validation studies, dissertations, narrative, scoping, and systematic reviews, and articles that are not primary research studies will be excluded.

The population of interest for this scoping review is NGNs. The context of interest is the first year of practice, comprising the early practice period. Articles that involve participants with 0–12 months of nursing experience will be included. The first year of practice is used to theoretically align with the Stages of Transition model [12]. Articles that focus on nursing students, nurses with more than 12 months of experience or non-nurse participants will be excluded, as the purpose of these technologies is teaching and learning, not providing nursing care. NGNs categorized as a subgroup in a study will be included if the data can be extracted independently from other study participants. Studies that involve non-nurse, licensed practical nurses, registered practical nurses, and nurse practitioners will be excluded. Detailed eligibility criteria are outlined in Supplementary Material S1.

The key concept is digital health technologies, where digital health is defined as “the field of knowledge and practice associated with the development and use of digital technologies to improve health” [1]. Articles that focus on technology for teaching and learning or patient-oriented technology will be excluded.

Articles published within the last five years will be included. Articles published before 2020 will be excluded to ensure homogeneity in the digital health technologies included or used in the findings. The year of publication will be extracted for included articles, and the spread of publications can be examined for trends to contextualize the findings.

### 3.2. Search Strategy

The search strategy has been developed in consultation with the University of Ottawa librarian and will use key articles to verify the strategy [6,14]. A search of CINAHL, MEDLINE, PsycINFO, Embase, and ERIC will be conducted based on a comprehensive search strategy developed in consultation with the librarian. The search concepts are: “new graduate nurse” and “digital health”. The following search syntax is summarized: “Digital health” or “digital health technology” or “nursing informatics” or “virtual nursing” or “health informatics” or “medical informatics” or “e-health” or “m-health” AND “new nurses” or “new graduate” or “entry-to-practice” or “entry level” or “new nurse” or “new clinician” or “graduate nurse” or “newly registered” or “newly employed nurse” or “novice nurse”.

## 4. Data Collection

Included articles will be added to the Covidence systematic review software (Veritas Health Innovation, Melbourne, Australia). The first phase of screening will then take place according to the title and abstract by the author and one other person who has content experience and has undergone education on the eligibility criteria, including piloting to ensure accuracy in screening. In the event of disagreements during the screening process, screeners will engage in a conversation where the rationale is provided. If this does not result in a resolution of the disagreement, a third screener will decide whether the article is included or not. Next, the second phase will consist of full-text screening of included articles, followed by data extraction from the final articles using an iterative process as per JBI guidance [13]. Phase two screening will involve two independent screeners, as in phase one. The process of disagreements will follow the same steps as the title and abstract screening phase. Data extraction (Supplementary Material S3) will occur independently by the researcher using the data extraction tool, which will be piloted to ensure usability and familiarity [13]. This tool includes the article authors, year of publication, country of origin, aim, sample, methodology, education intervention type, outcome and other key findings that relate to the research question [13]. The data extraction tool was created using the JBI guidance for scoping reviews to include key study demographic data, follow the population, context, and concept framework and key data extraction points related to the research question. A critical appraisal of the included articles will not be included as it is not required for a scoping review [13].

## 5. Analysis Plan

Data analysis will occur in accordance with the JBI guidance [13]. Data is reported in tables and charts as outlined in the Preliminary Data Extraction tool (Supplementary Material S4). Basic descriptive statistics, including frequencies and percentages or proportions, will be used to quantify the data [13]. Qualitative analysis involves a basic descriptive approach, where data is categorized using simple coding [13]. A narrative synthesis format will be used to clarify concepts and definitions that emerge from the data extraction, which may include flow diagrams and concept maps. Finally, a narrative discussion section will articulate the findings and outline the limitations and implications of the scoping review [13].

## 6. Conclusions

The results of this scoping review will elucidate the literature available that describes and explores NGNs and digital health technologies. Potential limitations of this review include limited primary research studies that meet the inclusion criteria, heterogeneity of articles, and the possibility of excluding articles published before 2020. Despite these potential limitations, articulating the available literature on digital health and NGNs can enhance patient care by building the foundational knowledge required to empower NGNs to leverage digital health technology in the provision of care. Findings from this scoping review will be of interest to nurse educators, NGNs and clinical practice policy makers and educators who work with NGNs. Exploring the implications of digital health technology integration for NGNs in this scoping review is foundational for future research as the nursing profession continues to evolve.

## Data Availability

No new data were created or analyzed in this study.

## Supplementary Materials

The following supporting information can be downloaded at: https://www.mdpi.com/article/10.3390/nursrep16030090/s1, Supplementary Material S1: Preliminary Scoping Review Eligibility Criteria; Supplementary Material S2: Search Strategy; Supplementary Material S3: PRISMA: ScR [15]; Supplementary Material S4: Scoping Review Data Extraction Tool.

## Public Involvement Statement

No public involvement in any aspect of this research.

## Guidelines and Standards Statement

This manuscript was drafted using the PRISMA-ScR checklist for reporting scoping reviews.

## Abbreviations

The following abbreviations are used in this manuscript:

NGN	New graduate nurse
JBI	Joanna Briggs Institute
