# Supplementary material for: Digital Health Technology and the New Graduate Nurse: A Scoping Review Protocol
Source: Nurs Rep. 2026 Mar 5;16(3):90. doi: 10.3390/nursrep16030090 (PMC13029048; doi:10.3390/nursrep16030090)
Supplement: Supplementary file 1 [file nursrep-16-00090-s001.zip › nursrep-4153972-supplementary.pdf]

Supplementary Material S1. Preliminary Scoping Review Eligibility Criteria

Eligibility Criteria:

	Inclusion	Exclusion
P	New graduate nurses (0–12 months of experience)<br>New graduate nurses can be included as a subgroup of a study with other professionals if the participant data is clearly reported separately from others.	Participants who are not new graduate nurses within the first year of practice, including nurse practitioners, registered practical nurses, licensed practical nurses<br>Non-nurse participants<br>Nursing students<br>Studies where NGN data is not separately reported
C	Definition: "the field of knowledge and practice associated with the development and use of digital technologies to improve health."(World Health Organization, 2021, p.11).	Technology that falls outside of this definition.<br>Educational sessions that are a part of the nursing curriculum.
C	Settings can include acute care of patients of any age, in hospital or community-based, rural, remote, or urban settings. Any nursing specialty. In an orientation program or a new graduate nurse focus education program.<br>Findings must explore any dimension of transition to practice in the first year of practice.	
Other	Studies must include (peer-reviewed, quantitative, qualitative or mixed-methods studies) digital health technology.	Tool-validation studies. Dissertations, gray literature, narrative, scoping and systematic reviews, and articles that are not primary research studies

Supplementary Material S2. Search Strategy

Digital Health Technology		New Graduate Nurse
Digital health OR technology OR nursing informatics OR virtual nursing OR health informatics OR medical informatics OR e-health OR m-health OR remote health	AND	New nurses OR new graduate OR entry-to-practice OR entry level OR new nurse OR new clinician OR graduate OR newly registered OR licensed OR employed nurse OR novice nurse

Key Articles: Kaihlanen et al., (2021) and Kleib et al., (2023)

Search Strategy Example: CINAHL

Line	Query	Results
1	(MH "Nursing Informatics") OR (MH "Health Informatics") OR (MH "Digital technology") OR (MH "Digital health") OR ("Artificial Intelligence")	44,371
2	Tl (digital N3 (health or care or healthcare or consult * or visit * or nursing)) OR AB (digital N3 (health or care or healthcare or consult* or visit * or nursing))	4789
3	Tl (virtual N3(health or care or consult * or visit * or nurs * or monitor *)) OR AB (virtual N3(health or care or consult * or visit * or nurs * or monitor *))	3636
4	Tl (eHealth or e-health or mhealth or m-health or (mobile N2(health or care or healthcare or consult * or nursing or app # or application *)) OR AB (eHealth or e-health or mhealth or m-health or (mobile	49,199

	N2(health or care or healthcare or consult * or nursing or app # or application *))	
5	Tl ((nursing or health)N2 informatics) OR AB ((nursing or health)N2 informatics))	1622
6	Tl (tele N2(health or care or consult * or visit * or nurs * or monitor)) OR AB (tele N2(health or care or consult * or visit * or nurs * or monitor))	506
7	Tl ("artificial intelligence" or AI or robot * or "voice recognition") OR AB ("artificial intelligence" or AI or robot * or "voice recognition")	26,137
8	S1 OR S2 OR S3 OR S4 OR S5 OR S6 OR S7	111,714
9	(MH "new graduate nurses") OR (MH "Novice Nurses")	9137
10	Tl (nurse * N2(new or novice or young or register * or begin * or graduat *)) OR AB (nurse * N2(new or novice or young or register* or begin * or graduat *))	24,021
11	Tl (nurs * N3(begin * or entry-to-practice or newly employed or newly licensed)) OR AB (nurs * N3(begin * or entry-to-practice or newly employed or newly licensed))	1624
12	S9 OR S10 OR S11	31,275
13	(S9 OR S10 OR S11) AND (S8 AND S12)	527

Supplementary Material S3. PRISMA: ScR

Preferred Reporting Items for Systematic reviews and Meta-Analyses extension for Scoping Reviews (PRISMA-ScR) Checklist

SECTION	ITEM	PRISMA-ScR CHECKLIST ITEM	REPORTED ON PAGE #
**TITLE**			
Title	1	Identify the report as a scoping review.	Page 1
**ABSTRACT**			
Structured summary	2	Provide a structured summary that includes (as applicable): background, objectives, eligibility criteria, sources of evidence, charting methods, results, and conclusions that relate to the review questions and objectives.	1
**INTRODUCTION**			
Rationale	3	Describe the rationale for the review in the context of what is already known. Explain why the review questions/objectives lend themselves to a scoping review approach.	1-2
Objectives	4	Provide an explicit statement of the questions and objectives being addressed with reference to their key elements (e.g., population or participants, concepts, and context) or other relevant key elements used to conceptualize the review questions and/or objectives.	2
**METHODS**			
Protocol and registration	5	Indicate whether a review protocol exists; state if and where it can be accessed (e.g., a Web address); and if available, provide registration information, including the registration number.	3
Eligibility criteria	6	Specify characteristics of the sources of evidence used as eligibility criteria (e.g., years considered, language, and publication status) and provide a rationale.	3

Information sources *	7	Describe all information sources in the search (e.g., databases with dates of coverage and contact with authors to identify additional sources), as well as the date the most recent search was executed.	3
Search	8	Present the full electronic search strategy for at least 1 database, including any limits used, such that it could be repeated.	S1
Selection of sources of evidence †	9	State the process for selecting sources of evidence (i.e., screening and eligibility) included in the scoping review.	3-4
Data charting process ‡	10	Describe the methods of charting data from the included sources of evidence (e.g., calibrated forms or forms that have been tested by the team before their use, and whether data charting was done independently or in duplicate) and any processes for obtaining and confirming data from investigators.	4
Data items	11	List and define all variables for which data were sought and any assumptions and simplifications made.	N/A
Critical appraisal of individual sources of evidence §	12	If done, provide a rationale for conducting a critical appraisal of included sources of evidence; describe the methods used and how this information was used in any data synthesis (if appropriate).	N/A
Synthesis of results	13	Describe the methods of handling and summarizing the data that were charted.	4
**RESULTS**			
Selection of sources of evidence	14	Give numbers of sources of evidence screened, assessed for eligibility, and included in the review, with reasons for exclusions at each stage, ideally using a flow diagram.	Click here to enter text.
Characteristics of sources of evidence	15	For each source of evidence, present characteristics for which data were charted and provide the citations.	Click here to enter text.
Critical appraisal within sources of evidence	16	If done, present data on critical appraisal of included sources of evidence (see item 12).	Click here to enter text.
Results of individual sources of evidence	17	For each included source of evidence, present the relevant data that were charted that relate to the review questions and objectives.	Click here to enter text.
Synthesis of results	18	Summarize and/or present the charting results as they relate to the review questions and objectives.	Click here to enter text.
**DISCUSSION**			
Summary of evidence	19	Summarize the main results (including an overview of concepts, themes, and types of evidence available), link to the review questions and objectives, and consider the relevance to key groups.	N/A
Limitations	20	Discuss the limitations of the scoping review process.	4
Conclusions	21	Provide a general interpretation of the results with respect to the review questions and objectives, as well as potential implications and/or next steps.	4
**FUNDING**			
Funding	22	Describe sources of funding for the included sources of evidence, as well as sources of funding for the scoping review. Describe the role of the funders of the scoping review.	5

JBI = Joanna Briggs Institute; PRISMA-ScR = Preferred Reporting Items for Systematic reviews and Meta-Analyses extension for Scoping Reviews. * Where *sources of evidence* (see second footnote) are compiled from, such as bibliographic databases, social media platforms, and websites. † A more inclusive/heterogeneous term used to account for the different types of evidence or data sources (e.g., quantitative and/or qualitative research, expert opinion, and policy documents) that may be eligible in a scoping review as

opposed to only studies. This is not to be confused with *information sources* (see first footnote). ‡ The frameworks by Arksey and O'Malley (6) and Levac and colleagues (7) and the JBI guidance (4, 5) refer to the process of data extraction in a scoping review as data charting. § The process of systematically examining research evidence to assess its validity, results, and relevance before using it to inform a decision. This term is used for items 12 and 16 instead of "risk of bias" (which is more applicable to systematic reviews of interventions) to include and acknowledge the various sources of evidence that may be used in a scoping review (e.g., quantitative and/or qualitative research, expert opinion, and policy document).

From: Tricco AC, Lillie E, Zarin W, O'Brien KK, Colquhoun H, Levac D et al. PRISMA Extension for Scoping Reviews (PRISMAScR): Checklist and Explanation. Ann Intern Med. 2018;169:467–473. doi: 10.7326/M18-0850.

Supplementary Material S4. Scoping Review Data Extraction Tool

Review Objective:

The objective of this review is to explore and describe the literature about digital health technologies and new graduate nurses.

Question:	Guiding Information	Study Data
	**Eligibility Criteria:**	
**Population**	Must involve new graduate nurses, from zero to twelve months of experience	
**Concept**	Digital health type (what technology(ies) does the study describe?)	
**Context**	Practice setting	
**Type of evidence/study**	Primary research: mixed-methods, quantitative or qualitative	
	**Evidence Description:**	
**Citation details**	Authors, date, title, journal	
**Country/Region**		
**Context**	(clinical setting, unit)	
**Instrument/tool and psychometric properties**	If used	
**Participants description**	Number, age, gender, orientation length	
	**Details/Results Extracted**	
**Study context**	(describe study setting, context, discusses proficiency of participants)	
**Digital Health technology**	Describe technology used in study.	
**Links to transition to practice**	How does the outcome of the study link to transition to practice, for example, is digital technology a facilitator or barrier<br>Is there an implication for proficiency?	
**Other**	Other key findings	
